# Epitope Mapping of *Streptococcus agalactiae* Elongation Factor Tu Protein Recognized by Human Sera

**DOI:** 10.3389/fmicb.2018.00125

**Published:** 2018-02-06

**Authors:** Marcelina Pyclik, Sabina Górska, Ewa Brzozowska, Anna Dobrut, Jarosław Ciekot, Andrzej Gamian, Monika Brzychczy-Włoch

**Affiliations:** ^1^Ludwik Hirszfeld Institute of Immunology and Experimental Therapy, Polish Academy of Sciences, Wroclaw, Poland; ^2^Chair of Microbiology, Department of Molecular Medical Microbiology, Jagiellonian University Medical College, Krakow, Poland

**Keywords:** *Streptococcus agalactiae*, group B streptococcus, elongation factor Tu, epitope mapping, vaccination, synthetic peptide, PEPSCAN, ELISA

## Abstract

The elongation factor Tu has been identified as one of the most immunoreactive proteins that was recognized by human sera of GBS (group B streptococcus) positive patients. In this paper, we present the polypeptide-specific epitopes of the bacterial protein that are recognized by human antibodies: ^28^LTAAITTVLARRLP^41^ (peptide no. 3) and ^294^GQVLAKPGSINPHTKF^309^ (peptide no. 21). To determine the shortest amino acid sequence recognized by antibodies, truncation peptide libraries were prepared using the PEPSCAN method. The analysis of immunoreactivity of peptides with sera of GBS positive and negative women revealed that the most immunoreactive sequence was ^306^HTKF^309^. Moreover, we observed that this sequence also showed the highest specificity which was based on ratio of reactivity with sera of GBS positive relative to sera of GBS negative patients. Epitope was synthetized on Wang resin with the Fmoc strategy. Our results open the possibility to use ^306^HTKF^309^ peptide in diagnostic assays to determine *Streptococcus agalactiae* infection in humans.

## Introduction

*Streptococcus agalactiae* (group B streptococcus, GBS) is a Gram-positive bacterium that can colonize human gastrointestinal and genitourinary tracts without any symptoms of diseases. Ten to thirty percent of pregnant women are estimated to be colonized with GBS (Johri et al., 2013), which can be dangerous for newborn children. In south-eastern Poland, the colonization with *S. agalactiae* among pregnant women amounts to 30%, and the increase in the number of infections in term newborns reaches 0.15 per 1,000 live births, whereas in the preterm newborn incidence of infection increase to 6 cases per 1,000 live births (Brzychczy-Włoch et al., 2013a,b; Centers for Disease Control and Prevention [CDC], 2015). In the 1980s, to decrease the risk of GBS transmission to newborns, administration of antibiotic during labor of GBS positive women was recommended by the American College of Obstetricians and Gynecologists. Schrag et al. (2002) guideline recommended the antenatal screening approach or evaluation of risk factors at the time of delivery for the administration of antibiotic intrapartum prophylaxis. Only after revision of many additional data, CDC opted for universal antenatal screening as the only option for the prevention of neonatal GBS disease (Centers for Disease Control and Prevention [CDC], 2010 guideline). The methodology of GBS detection is based on screening by vaginal-rectal swabbing. To identify GBS isolates, screening with selective enrichment broth is used and the results are known in about 48 h (Centers for Disease Control and Prevention [CDC], 2010). Other methods for GBS identification are as follows: CAMP test (Ratner et al., 1986), serologic test (Guerrero et al., 2004), test on chromogenic agars (non-hemolytic GBS is not detected; Votava et al., 2001), or DNA analysis (Ryan et al., 1999; Goodrich and Miller, 2007). Rapid diagnostics for carriage/infection in pregnant women, especially those giving birth prematurely, would guarantee immediate implementation of antibiotic prophylaxis/therapy. When considering intermittent colonization with GBS, antenatal screening can give false negative results which lead to the occurrence of Early Onset Desease (EOD) in 20–30% of cases in newborns delivered by pregnant women who were diagnosed as healthy (Berardi et al., 2013; Schrag and Verani, 2013). A rapid immunosorbent assay which is very sensitive and specific and is able to show specific protective antibodies against GBS antigens could be used to verify the fact that the pregnant women indeed had been exposed to GBS. The rapid immunosorbent assay could also be used in situations when the pregnant women had not been subjected to antenatal screening, e.g., in premature labor. New DNA-based rapid tests are not available in many countries, what is affected by high costs of analysis and requires expensive apparatus. Moreover, most of accessible molecular methods are standardized to vaginal colonization diagnosis, while there are no procedures for molecular identification of GBS in rectum, what can lead to false negative results.

In our previous studies, elongation Tu factor was shown as a highly immunoreactive protein that can be used as marker of GBS infection and/or colonization (Brzychczy-Włoch et al., 2013c). In this study, we would like to determine the most specific epitope of the protein and check it as a marker of GBS infection in enzyme-linked immunosorbent assay (ELISA) with human GBS positive as well as GBS negative sera. The epitopes were selected using PEPSCAN methodology. This technique allows to synthesize many peptides in one tray linked to plastic pins and then examine them in ELISA.

## Materials and Methods

### Study Population and Specimen Collection

The study group (*n* = 20) comprised pregnant women with confirmed colonization by *S. agalactiae* according to CDC recommendation (Centers for Disease Control and Prevention [CDC], 2010). The control group (*n* = 14) consisted of pregnant women not colonized with *S. agalactiae.*

The inclusion criteria:

- Pregnant women in the third trimester between 18 and 40 years of age
- A written statement of consent to participate in the study

The exclusion criteria:

- Pregnant women below 18 and over 40 years of age
- Patients with immunodeficiency or autoimmune diseases
- Pregnant women with the so-called high-risk pregnancy or with perinatal complications
- Preterm delivery
- No written consent to participate in the study or its withdrawal

The screening for GBS carriage in pregnant women was performed at 35–37 weeks gestation. The swabs were collected from the lower vagina (vaginal introitus), followed by the rectum (i.e., inserted swab through the anal sphincter). Swabs were taken separately by two sterile cotton swabs during the standard pelvic examination in the third trimester of pregnancy. The procedure for culture and identification of GBS in the studied materials were conducted in accordance with the CDC recommendations (Centers for Disease Control and Prevention [CDC], 2010). Samples of approximately 50 ml of umbilical cord blood were collected after the safe completion of delivery of patients included in our investigation (*n* = 34), both from study group (*n* = 20) and control group (*n* = 14) and were used to obtain serum. Serum samples were stored at -70°C in order to conduct immunoassays on protein antigens of GBS. In addition, serum samples from venous blood collected in the third trimester of pregnancy from pregnant women colonized with GBS (*n* = 10), serum samples collected in the third trimester of pregnancy from pregnant women without GBS colonization (*n* = 8) and serum from neonates with GBS EOD sepsis (*n* = 2) collected in the course of the project number NN401042337 and described in our previous paper were used (Brzychczy-Włoch et al., 2013c). The study was approved by the Jagiellonian University Bioethical Committee decision no. KBET/153/B/2014.

### Bacterial Strains

*Streptococcus agalactiae* isolates (*n* = 120) were drawn from the study group of pregnant women (*n* = 20), as well as from the urinary tract infections (UTI; *n* = 100). The aim of the inclusion of bacterial strains isolated from various infections types was identification of common immunoreactive proteins, which could be universal infection marker. The GBS strains from UTI were isolated from urine samples from patients with *S. agalactiae* bacteriuria ≥1 × 10^5^ cfu/ml and were collected in the years 2009–2013 as part of the current diagnostics of the Microbiology Laboratory of the Chair of Microbiology Jagiellonian University Medical College in Krakow. The identification of GBS based on PCR with species-specific primers was applied (Ke et al., 2000). The molecular characterization of the tested GBS strains was carried out according to the method described in our previous publication (Brzychczy-Włoch et al., 2013c).

### Protein Isolation and Analysis

Proteins were isolated and analyzed by slight modification of a previously described procedure (Brzychczy-Włoch et al., 2013c). Briefly, bacteria were cultured on brain heart infusion broth (Biocorp) at 37°C for 24 h. After centrifugation, they were suspended directly in the buffer for electrophoresis according to Heilmann et al. (1996). This made it possible to isolate moonlighting proteins and preserve their native structure. Protein concentration was analyzed using the Lowry’s method (Lowry et al., 1951). The immunoreactive proteins were identified by immunoblotting within a complex protein mixture that has been fractionated using discontinuous gradient polyacrylamide gel (5, 7.5, 10, and 12.5%). Individual reactive spots were cut out from gels and submitted to tryptic digestion, then analyzed by mass spectrometry (spectrometer LC-MS/MS Orbitrap, Thermo). Proteins were identified by Mascot^1^ (Matrix Science, London, United Kingdom) and statistical methods.

### Bioinformatic Analysis of *Streptococcus agalactiae* Elongation Factor Tu

Predictions of epitope regions were made based on sequence analysis. We used Genesilico Metaserver^2^ for predictions of secondary structure elements and loop regions, disordered regions, and protein solvation. Additionally, we used web server for epitope localization prediction: Antibody Epitope Prediction^3^ applied methods—Emini Surface Accessibility Prediction (Emini et al., 1985), Kolaskar & Tongaonkar Antigenicity (Kolaskar and Tongaonkar, 1990) and Bepipred Linear Epitope Prediction (Larsen et al., 2006), as another cross method BCPREDS was used from B-cell epitope prediction server^4^. For more detailed predictions, we used homology models made based on 3D structure of PDB: 2C78 [elongation factor Tu (EF-TU) complexed with a GTP analog and the antibiotic pulvomycin]. Based on the 3D structure of the modeled protein, we identified loop regions on the surface of protein with tendency to be disordered and which could be a good epitope. The results obtained were used to make consensus predictions. Any sequences that were positively selected by more than two prediction methods and that contained more than six amino acids were selected as linear epitopes.

### Synthesis of Peptides Tethered to Polyethylene Pins

Peptides corresponding to predicted epitopes were synthesized using NCP Block of 96 hydroxypropyl methacrylate pins (MIMOTOPES, Clayton, VIC, Australia) by stepwise elongation of the peptide chain from the C- to the N-terminus, according to the procedure described by Geysen et al. (1987) with slight modifications. Each pin was suspended in 100 μl of the solution containing 60 mM Fmoc amino acid and equimolar amounts of diisopropylcarbodiimide (DIC) and 1-hydroxy-7-azabenzotriazole (HOAt). Each coupling cycle lasted 6 h. Finally, peptides were deprotected, washed with methanol (MeOH), then with 0.5% acetic acid in MeOH and again with MeOH and dried. Peptides were stored at -20°C before use.

### The Enzyme-Linked Immunosorbent Assay (ELISA) with Peptides on Pins

Pins were disrupted in the buffer composed of 1% SDS, 0.1% 2-mercaptoethanol, and 0.1 M Na_3_PO_4_, pH 7.2, heated to 60°C and placed in a sonication bath for 10 min. Pins were rinsed with water and placed in a 60°C water-bath for 5 min and then in hot MeOH for 2–3 min. Before ELISA pins were equilibrated with TBST (PBS + 0.05% Tween) for 10 min. at 25°C. Pins were transferred to flat-bottom 96-well MaxiSorp plates (Nunc) filled with 200 μl of 1% bovine serum albumin (BSA) in TBST for 1 h at room temperature. They were then immersed in 100 μl of the human umbilical cord serum (1:1,000) and incubated for 2 h at 25°C. Following three washes, the alkaline phosphatase-conjugated goat anti-human IgG (1:10,000; ICN Biomedicals, Aurora, OH, United States) were applied and the plates were incubated at 25°C for 1 h. The level of bound conjugate was indicative of peptide antigenic activity and was measured through the alkaline phosphatase activity. After five washing steps with TBST, 200 μl of the *p*-nitrophenyl phosphate (pNPP; liquid substrate for ELISA, Sigma) was used. To stop the enzymatic reaction, pins were removed from the plate and released *p*-nitrophenol was measured at 405 nm on a BioTek Instruments (Tecan). To re-use pins they were disrupted and stored at -20°C with silica gel.

### Solid Phase Peptide Synthesis

Synthesis of the polypeptides was performed on Wang resin with the Fmoc strategy. Hundred milligrams of the resin was incubated in 2 ml of *N*-dimethylformamide (DMF) for 1 h. The resin activation was performed using 2 ml of 20% (v/v) piperidine (PIP) dissolved in DMF, then it was washed six times with DMF. A 0.5 mmol (5 equiv) of each Fmoc amino acid was used in the elongation reaction as well as 43.7μl of HOAt and 43.9 μl of DIC reagents. The elongation time was varying from 6 to 36 h depending on the amino acid residue. Next, the solvent was drained and the resin was washed with DMF (6 × 2 ml). The Fmoc protecting group was removed with a solution of PIP/DMF (2/8, 2 ml) for 30 min (deprotection). The solution was drained and the resin was washed with DMF. After the last deprotection step, the resin was washed with DMF (6 × 2 ml), dichloromethane (2 × 2 ml), and MeOH (2 × 2 ml). After last washing, the resin was dried under vacuum. The peptides were cleaved from the resin with trifluoroacetic acid (TFA; 95%) and precipitated with cold diethyl ether. Next they were centrifuged (4,000 × *g*, 10 min × 2), dissolved in water and lyophilized. They were stored at -20°C.

### The Purification of the Peptide

Sample preparation was performed by dissolving in a mixture of water, acetonitrile, and formic acid (94.9, 5, 0.1% v/v/v). Immediately before HPLC analysis, samples were filtered through a 0.22 μm filter. The apparatus used was Dionex Ultimate 3000 System (Dionex Corporation, United States) equipped with LPG-3400SD pump, WPS-3000T(B) FC Analytical autosampler, TCC-3000SD column compartment and a DAD-3000 diode array detector variable. The chromatography column was a C18 column (Phenomenex, 250 mm × 4.6 mm, 5 μm, 110 Å). The following chromatographic conditions were applied: the mobile phase: solvent A—0.1% formic acid in water; solvent B—0.1% formic acid in acetonitrile. The program was as follows: 0 min—5% solvent B; 15 min—95% solvent B; 16 min—95% solvent B; 16.3 min—5% solvent B; 27.5 min—5% solvent B. The inject sample volume was 100 μl. The flow rate was 0.5 ml/min and the eluent was monitored at 254 nm at room temperature.

### Analysis of the Peptide after Purification Process

Samples from the purification process were lyophilized and then dissolved in the mixture of water, acetonitrile, and formic acid (94.9, 5, 0.1% v/v/v). Immediately before HPLC, samples were filtered through a 0.22 μm filter and analyzed on apparatus Dionex 3000 RS-HPLC equipped with a DGP-3600 pump, a WPS-3000 TLS TRS autosampler, a TCC-3000 RS column compartment and a DAD-3000 RS diode array detector variable (Dionex Corporation, United States). The chromatography column was a 50 × 3.1 (i.d)-millimeter Thermo Scientific Hypersil GOLD with 1.9-micron particles (Part no. 25002-052130). The following chromatographic conditions were applied: the mobile phase: solvent A—0.1% formic acid in water; solvent B—0.1% formic acid in acetonitrile. The program was as flows: 0 min—5% solvent B; 1 min—5% solvent B; 21 min—95% solvent B; 21.5 min—95% solvent B; 21.6 min—5% solvent B; 30 min—5% solvent B. The inject sample volume was 4 μl. The flow rate was 0.4 ml/min and the eluent was monitored at 254 nm at room temperature. The peptide was analyzed in MALDI-TOF-MS using α-cyano-4-hydroxycinnamic acid as a matrix. Five microliters of the peptide solution (1 mg/ml) was mixed with 5 μl of the matrix (1 mg/ml in 80% acetonitrile containing 1% TFA).

### ELISA with the Peptide Coated on Plate

A 96-well MaxiSorp plate (NUNC, United States) was coated with 10 μg/well of the peptide in coating buffer (10 mM NaHCO_3_ buffer, pH 9.6) and incubated at 4°C overnight. The plate was washed three times with phosphate buffered saline containing 0.05% Tween-20 (PBST) after each incubation. Peptide-coated plate was blocked by incubation with 200 μl blocking buffer (1% BSA in PBST) at 37°C for 1 h, and incubated with 100 μl of human sera (1:1,000 dilution) in blocking buffer at 37°C for 45 min. Following three washes, the alkaline phosphatase-conjugated goat anti-human IgG (1:10,000; ICN Biomedicals, Aurora, OH, United States) were applied and the plates were incubated at 25°C for 1 h. The level of bound conjugate was indicative of peptide antigenic activity and was measured through the alkaline phosphatase activity. After five washing steps with TBST, 200 μl of the pNPP (liquid substrate for ELISA, Sigma) was used. To stop the enzymatic reaction, 2 M NaOH was added and measured at 405 nm on a BioTek Instruments (Tecan).

### Statistical Analyses

Statistical analysis was performed by one-way analysis of variance followed by Tukey’s multiple comparison test using Prism 5.04 software (GraphPad, San Diego, CA, United States). *P*-values of <0.05 were considered significant. Data are expressed as means standard errors of the means (SEM).

## Results

Our previous studies revealed the existence of at least four immunoreactive proteins present in 60 studied strains of *S. agalactiae*, among which EF-Tu (44 kDa) was identified (Brzychczy-Włoch et al., 2013c). In the current studies, we expanded the studied test pool of GBS isolates by new genetically different clinical strains (*n* = 120) as well as the serum samples originating from umbilical cord blood of GBS-positive (GBS+, *n* = 20) and GBS-negative (GBS-, *n* = 14) patients. Proteins from three GBS isolates were subjected for bioinformatics analysis, namely: (1) isolate no. D129 (sample from adult with UTI), III serotype and *rib* gene; (2) isolate no. 1736/08 (sample from neonate with UTI), serotype V, *alp*2 gene, cMLS_B_ phenotype, and *erm*B gene; (3) isolate no. 13793/08 (sample from neonate with UTI), serotype V, *alp*3 gene, cMLS_B_ phenotype, and *erm*B gene. Again, we identified that the EF-Tu exhibited the highest immunoreactivity. To predict the epitope regions the bioinformatics analysis based on amino acid sequence was made. Moreover, based on the 3D structure of the modeled protein, we identified loop regions on the surface of protein with a tendency to be disordered and which could be a good epitope. In general, we projected 28 potential epitopes (**Table 1**).

**Table 1:** List of the predicted synthetic epitopes of *S. agalactiae* elongation factor Tu.

No.	Predicted epitope	No.	Predicted epitope
1	KEKYDRSKPHVNIG (3–16)	15	MSTVDEYIPEPERDTDK (196–212)
2	IGHVMH (18–23)	16	KPLLLPVEDVFSI (212–224)
3	LTAAITTVLARRLP (28–41)	17	RGTVASGRIDRGTVRV (227–243)
4	TSVNQPKDYASIDA (42–55)	18	VNDEVEIVGIK (244–252)
5	APEERERGITINTA (56–69)	19	QKAVVTGVEMFRKQLDEGLAGD (256–277)
6	HVEYETEKRH (70–79)	20	GDNVGVLLRGVQRDEIE (276–292)
7	HYAHIDAPGHADY (79–91)	21	GQVLAKPGSINPHTKF (294–309)
8	VKNMITGAAQM (92–102)	22	GEVYILSKEEGGRHTP (311–326)
9	GAILVVASTDGPMPQTR (104–120)	23	PFFNNYRPQFY (326–335)
10	TREHILLSR (119–127)	24	DVTGSIELPAG (341–351)
11	RQVGVKHLIVFMNKVDLV (127–144)	25	TEMVMPGDNVTIEVE (352–366)
12	LLELVEMRDLLSEY (149–164)	26	TIEVELIHPIAVEQG (362–376)
13	KEKYDRSKPHVNIG (3–16)	27	MSTVDEYIPEPERDTDK (196–212)
14	IGHVMH (18–23)	28	KPLLLPVEDVFSI (212–224)

**FIGURE 1:**
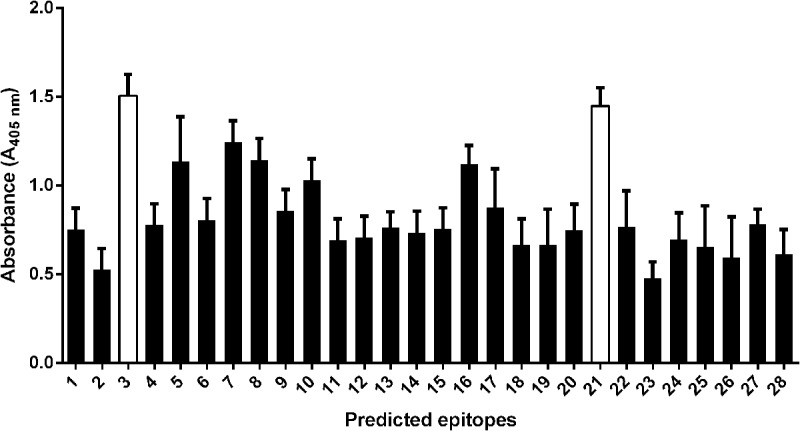
Recognition of the pin-tethered peptides representing predicted epitopes of *S. agalactiae* by human umbilical cord sera (pooled sera of GBS+) in ELISA. The empirical studies showed that two of the peptides were immunodominant in reaction with GBS+ sera (peptide no. 3 and 21). Experiments were performed in triplicate and results represent the mean of the Absorbance index ± SEM.

**FIGURE 2:**
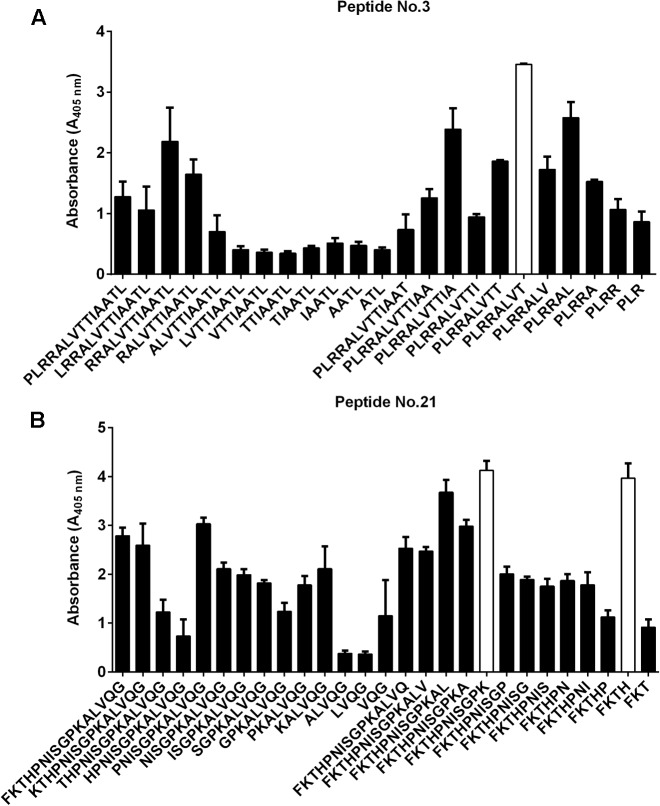
Reactivity of peptide no. 3 and its derivatives **(A)** as well as peptide no. 21 and its derivatives **(B)** with human umbilical cord sera (pooled sera of GBS+—in ELISA. The peptides (no 3 and no 21) were modified by removing the residues flanking of the N and C ends. Three epitopes were selected as the most immunoreactive: TVLARRLP—Ep1 **(A)**; KPGSINPHTKF—Ep2 and HTKF—Ep3 **(B)**. Experiments were performed in triplicates and results represent the mean of the Absorbance index ± SEM.

**FIGURE 3:**
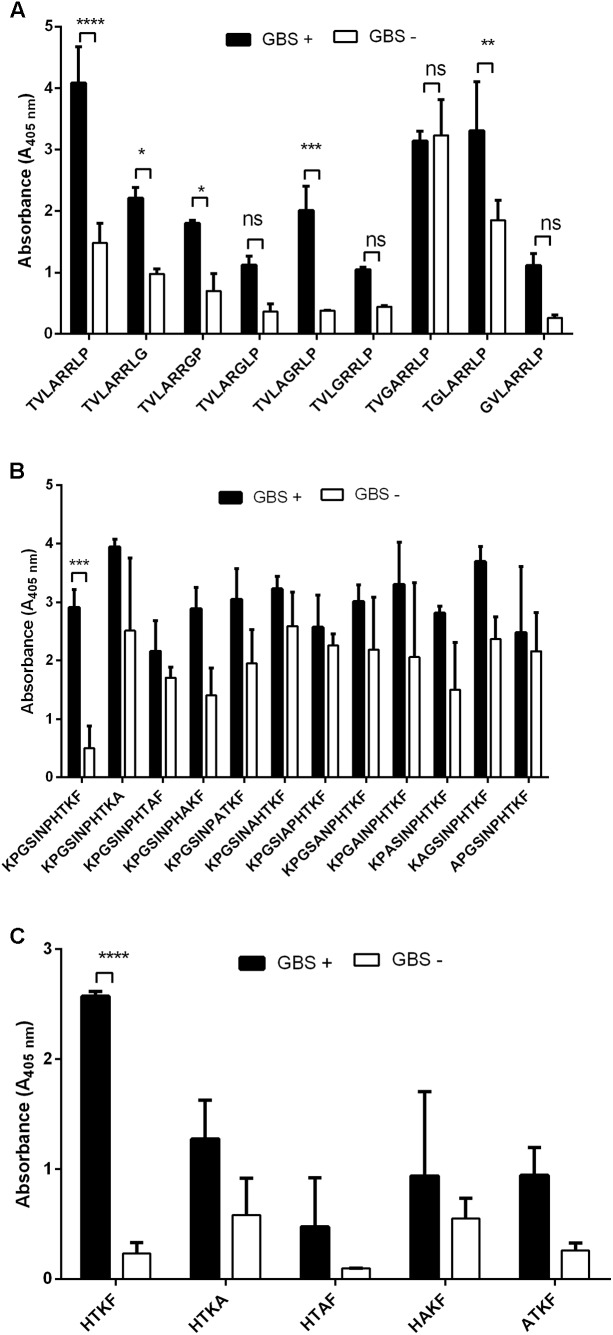
Reactivity of Ep1 **(A)**, Ep2 **(B)**, and Ep3 **(C)** and their derivatives with human umbilical cord sera (pooled GBS+ and pooled GBS– sera) in ELISA. Ep1 and Ep2 were modified by each amino acid substitution by glycine **(A)** or alanine **(B,C)**. The substitution of each amino acid by glycine in Ep1 or alanine in Ep3 caused the immunoreactivity decrease. In Ep, Thr, Ala, and two Arg, residues were the most crucial for the interaction with human antibodies, whereas in Ep2 and Ep3, the Lys residue was the most important for antibody binding. Experiments were performed in triplicates and results represent the mean of the absorbance index ± SEM. ^∗∗∗∗^*p* < 0.0001, ^∗∗∗^*p* < 0.001, ^∗∗^*p* < 0.01, ^∗^*p* < 0.05; ns, no significance.

To identify which of the projected epitopes are recognized by immunoglobulins, peptides (in the form of 6-mer to 20-mer peptides) bearing the sequence of the predicted epitopes were synthesized using pin technology and tested for their immunoreactivity with pooled human sera GBS- and GBS+. We used the pooled sera due to the limitation of reusability of pins in ELISA test. The ELISA results showed that two peptides: ^28^LTAAITTVLARRLP^41^ (peptide no. 3) and ^294^GQVLAKPGSINPHTKF^309^ (peptide no. 21) exhibited the highest immunoreactivity (**Figure 1**). To identify the shortest amino acid sequence necessary for recognition by the umbilical cord sera antibodies, we prepared the truncation peptide libraries. The libraries were constructed by systemically removing the residues flanking of the N and C ends from the original peptides (**Figures 2A,B**). The reactive sera showed the highest reactivity with sequences ^35^TVLARRLP^41^ (a part of the peptide no. 3—Ep1) and ^299^KPGSINPHTKF^309^ (a part of peptide no. 21—Ep2) or only ^306^HTKF^309^ (a part of peptide no. 21—Ep3). To identify specific amino acid residues responsible for peptide’s activity the alanine/glycine scanning was performed. Each of the eight residues in Ep1 were systematically replaced by glycine, whereas each of the eleven or four residues in Ep2 or Ep3, were systematically replaced by alanine. We have shown that in the case of Ep1 the substitution of each amino acid by glycine caused the immunoreactivity decrease. However, Thr, Ala, and two Arg residues were the most crucial for the interaction with human antibodies. The reduction of the immunoreactivity of the peptide no. 21 derivatives was also observed. It was shown that the Lys residue was the most important for antibody binding (**Figure 3**).

**FIGURE 4:**
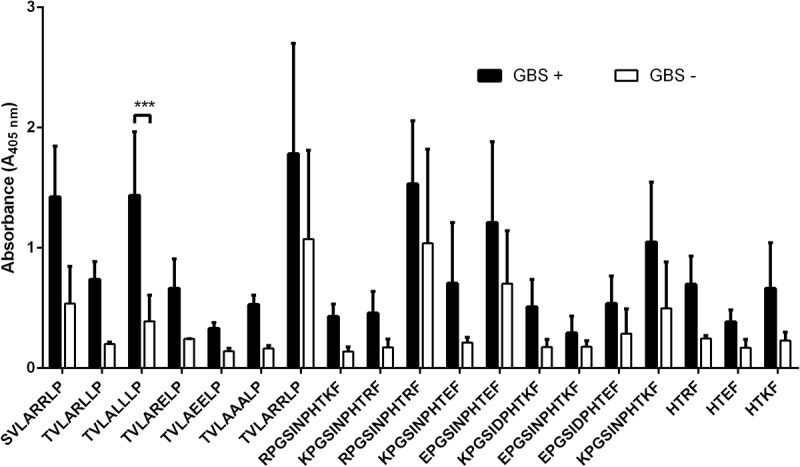
Reactivity of Ep1, Ep2, and Ep3 and their derivatives with human umbilical cord sera (pooled GBS+ and pooled GBS– sera) in ELISA. Ep1 was modified by substitution: Thr-Ser, Arg-Leu, ArgArg-AlaAla. Ep2 and Ep3 were modified by Lys-Arg or by Lys-Glu substitution. The substitution of one Arg with Leu or substitution of two Arg by Ala in Ep1 declined the absorbance for negative GBS and absorbance ratio. None of the modifications improved the parameters of the reactivity of the Ep2 and Ep3. Experiments were performed in triplicates and results represent the mean of the absorbance index ± SEM. ^∗∗∗^*p* < 0.001.

**FIGURE 5:**
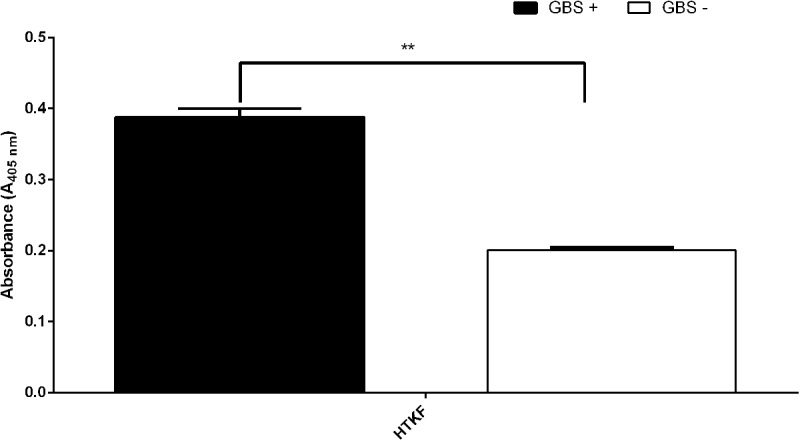
Reactivity of Ep3, with human umbilical cord sera (pooled GBS+ and pooled GBS– sera) in ELISA. The epitope showed the immunoreactivity with GBS+ serum, while the reactivity with GBS– serum was not observed. Experiments were performed in triplicates and results represent the mean of the absorbance index ± SEM. ^∗∗^*p* < 0.01.

To analyze the peptide reactivity with human sera three parameters were considered: the average absorbance at l = 405 nm with GBS+, the average absorbance with GBS- and their percentage ratio. Although Ep1 showed very high immunoreactivity with GBS+ sera, the reactivity with GBS- was also high. The average absorbance at l = 405 nm in ELISA for GBS- sera was 1.45 and represented 64% of the GBS+ absorbance (that indicated low specificity). To improve the specificity of the epitope, the Thr residue was replaced by Ser. The absorbance ratio of GBS+ and GBS- declined (35%), however, the absorbance of the negative sera was still high (0.71). The substitution of one Arg with Leu in this peptide—TVLARLLP—and substitution of two Arg by Ala residues—TVLAALP—declined these rates (absorbance for negative GBS and absorbance ratio) to 0.206 with 31% ratio and 0.16 with 28% ratio, respectively. Ep2 and Ep3 were modified by replacing the Lys residues by Arg or Glu. None of the modifications improved the parameters of the reactivity (**Figure 4**). All modifications of three epitopes are summarized in **Table 2** which showed the absorbance for GBS+, GBS-, and the absorbance ratio. Taking into account the limitations of the PEPSCAN method (peptides were immobilized on pins), we decided to synthesize the peptides in free form. We chose peptide ^306^HTKF^309^ due to the highest reactivity of sera from GBS positive patients. To check the immunoreactivity of the non-immobilized epitopes we synthesized them on Wang resin with the Fmoc strategy as described above. After chemical synthesis Ep3 was purified by HPLC and analyzed in MALDI as well as in ELISA with GBS+ and GBS- sera. The molecular mass of Ep3 was 616.64 Da and was about 85.036 Da higher than the calculated mass. The epitope showed the immunoreactivity with GBS positive serum that in fact was not as high as for epitope immobilized to pin. The average absorbance at l = 405 nm was 0.39, while the reactivity against GBS- serum was not observed (**Figure 5**). The average absorbance for GBS- was 0.21 and was identical as for the reagent sample. The percentage ratio of the absorbance GBS+ and GBS- was 54%. The experiment proved that the chosen epitope shows the specific immunoreactivity being immobilized as well as in the free form.

**Table 2:** Reactivity of modified epitopes with GBS+ and GBS- sera in ELISA.

	A_405__nm_ GBS+	A_405__nm_ GBS-	A_405__nm_ GBS+/A_405__nm_ GBS-
SVLARRLP	1.424	0.7155	50%
TVLARLLP	0.653	0.206	31%
TVLALLLP	1.437	0.511	35%
TVLARELP	0.523	0.244	46%
TVLAEELP	0.3575	0.14	39%
TVLAAALP	0.5735	0.16	28%
RPGSINPHTKF	0.43	0.139	32%
KPGSINPHTRF	0.458	0.132	29%
RPGSINPHTRF	1.8315	1.4625	79%
KPGSINPHTEF	0.706	0.246	35%
EPGSINPHTEF	1.5655	0.954	61%
KPGSIDPHTKF	0.51	0.14	27%
EPGSIDPHTEF	0.409	0.168	41%
HTRF	0.832	0.245	29%
HTEF	0.385	0.1305	34%
EPGSINPHTKF	0.292	0.177	60%
HTKF	**0.873**	**0.23**	**26%**
KPGSINPHTKF	**1.34**	**0.284**	**21%**
TVLARRLP	**2.3075**	**1.4795**	**64%**

*The absorbance (reactivity) value for unmodified peptides is marked*.

## Discussion

Currently, no quick diagnostic tests are available on the global market that would confirm the infection caused by *S. agalactiae*. Traditionally used diagnostic methods are based on culturing, the results of which are characterized by low sensitivity and long waiting periods (up to several days). Rapid diagnosis aimed at detecting carriers and infections caused by GBS guarantees immediate implementation of the targeted prevention or treatment. We identified specific epitopes of the bacterial protein EF-Tu that are recognized by human antibodies. EF-Tu is one of the most abundant and conserved bacterial proteins. It plays a central role in the elongation phase of protein synthesis and translation process in prokaryotic cells (Barbier et al., 2013). EF-Tu is a component of the bacterial membrane cytoskeleton involved in adhesion to the host cells in several pathogenic bacteria (Mayer, 2006) and was found also on the surface (Granato et al., 2004). EF-Tu is also known as a moonlighting protein that exhibits multiple biological functions involved in bacterial pathogenesis. It has been already shown that EE-Tu is involved in adhesion, invasion, and modulation of the host immune system (Kunert et al., 2007; Araujo et al., 2008; Mohan et al., 2014). In our previous study, the EF-Tu was identified as one of the most immunoreactive proteins that was recognized by human sera of GBS positive patients (Brzychczy-Włoch et al., 2013c). Other investigators have demonstrated that EF-Tu is one of the virulence factors of other bacterial infections. For example, Carrasco et al. (2015) demonstrated that EF-Tu is highly immunogenic in mice infected with *Borrelia burgdorferi* and in Lyme disease patients. Moreover, Jiang et al. (2016) suggested that EF-Tu may be a potential candidate for vaccine development against *Mycoplasma ovipneumoniae* infection in sheep. Recently, Widaja et al. (2017) indicated that EF-Tu moonlights on the surface of bacteria are a target of proteolytic processing events and suggested that studies are needed to determine how processing generates biologically important effector molecules and if protein processing is fundamental to the expansion of protein function in bacteria belonging to different phylogenetic clades.

Blast analysis indicates that the EF-Tu protein sequence from *S. agalactiae* is conserved. However, studies on moonlight proteins have shown that these proteins can change their spatial structure depending on the environment in which they are found. They may also be present in protein complexes, which may cause the exposure of other amino acid residues to the outer side of the protein in the case of membrane proteins (Jeffery, 2015). It was interesting to identify epitopes of this protein that evoke an immune response in GBS patients and open the possibility to use *S. agalactiae* EF-Tu as a potential antigen for serodiagnostic marker during GBS infection.

In this paper, we performed prediction analysis of presumable epitopes which are recognized by human antibodies with specific manner. The prediction was based upon amino acid sequence, additionally, we identified loop regions on the surface of the protein with a tendency to be disordered and which could be a good epitope candidate. This strategy allowed us to avoid the time-consuming continuous epitope analysis using Geysen procedure, which in case of EF-Tu (405 amino acid residues), would entail the combination (including overlapping peptides) of 8-mer peptides to result in about 400 combinations. Using bioinformatics analysis strategy, the total amount of projected epitopes were 28 (**Table 1**). The epitopes were synthesized on pins using the Fmoc strategy.

The empirical studies showed that two of the peptides were immunodominant in reaction with GBS+ sera (**Figure 1**). Another criterion for peptide selection was the lowest reactivity with GBS- sera, which is an evidence for the specificity of the peptides. Further modification of the original peptides gave us the shortest reactive epitopes with good specificity (**Figure 2**). In our study, as a background, we took the mean absorbance readings up to 30% of the mean absorbance obtained for positive sera for the same epitope. The goal of the immunoreactive peptide modification, namely cutting as well as amino acids replacing, was to obtain the epitopes as short as possible, but not shorter than four amino acids. The modification consisted in exchanging those amino acid residues that were most important for immunoreactivity (**Figure 3**). The important residues were substituted by amino acids containing similar physical properties: Thr was substituted by Ser, Arg by Leu, Lys by Arg. We also checked the immunoreactivity of Ep3 after Lys substitution by acidic amino acid—Glu (**Table 2**). None of the modifications caused immunoreactivity improvement except substitutions in Ep1, where one Arg was exchanged by Leu and two Arg by Ala residues (**Figure 4**). The immunoreactivity improvement should not surprise, because based on Kolaskar’s studies, Leu and Ala residues in general are often present in epitopes unlike the Arg residue.

Epitopes immobilized to pins can be reused only few times hence we used the PEPSCAN methodology only to determine the most specific epitopes against the pooled sera. The pooled sera consisted of umbilical cord blood sera of 15 individual patients that contained protected antibodies transferred from the mother’s blood. To analyze 100 or more human sera, we had to synthesize the epitope peptides and use them in the free form in ELISA. We decided to synthesize the shortest one of the epitope Ep3 and checked its reactivity. Ep3 was synthesized on resin using the standard methodology. After purification, we obtained 1 mg of Ep3 fragment peptide. Its molecular mass was higher than calculated (about 85.036 Da) which corresponds to molecular mass of PIP that was used during the synthesis procedure. We suggest that in the wake of the Thr residue a β-elimination reaction took place. The hydroxyl moiety was removed forming a dehydroalanine intermediate which reacts with the deprotecting base PIP, to form a 3-(1-piperidinyl)-alanine residue.

Ep3 was further analyzed in ELISA and its reactivity was not as high as in ELISA experiment with the same epitope immobilized to pin (**Figure 5**). The main reason of the difference could be insufficient physical adsorption of the free peptide to the plate. The short peptide could be washed out during ELISA performance. However, the methodology of the epitopes bounding to the plate (coating) should be improved. Linear epitopes should have from 5 to 22 amino acid residues (Andersen et al., 2006) or six to nine polar amino acids (Atassi, 1975), however, our Ep3 epitope is a tetrapeptide but exhibited the same reactivity as peptide no. 21. Moreover, we showed that each additional amino acid reduced its immunoreactivity, the specificity of this short sequence was 13 times higher than the modified sequence. The results obtained in the ELISA experiment give us a reason to expect that the two other epitopes would also be immunoreactive in the free form like Ep3.

The results of our studies show that to identify the presence of specific antibodies against *S. agalactiae* infection in humans we can use short, synthetic, specific epitopes that belong to elongation Tu factor. The protein is one of the most immunoreactive proteins of the *Streptococcus* strains that induce antibodies production in a natural way. The advantages of the epitopes are their high specificity, high yield and fast production process. Combination of two or three epitopes could further improve the diagnostic test based on the ELISA procedure. The epitopes could be also used as vaccine components, because, as we showed, the protective antibodies are transferred transplacentally from mother to child. However, we strongly emphasize that further, advanced research should be carried out.

## Author Contributions

MP carried out the protein isolation, peptide synthesis, and performed the ELISA. SG designed, coordinated, and conceived of the study, performed the protein identification, and drafted the manuscript. EB coordinated the solid phase peptide synthesis and purification, helped to draft the manuscript. AD performed the identification of bacterial strains and contributed reagents/materials. JC performed the purification using HPLC system. AG was a supervisor and helped to draft the manuscript. MB-W performed the study population and specimen collection, collected human sera, designed, coordinated, and helped to draft the manuscript. All authors read and approved the final manuscript.

## Conflict of Interest Statement

The authors declare that the research was conducted in the absence of any commercial or financial relationships that could be construed as a potential conflict of interest.

## Footnotes

1 http://www.matrixscience.com

2 https://genesilico.pl/meta2

3 http://tools.immuneepitope.org/

4 http://ailab.ist.psu.edu/
